# Effects of electroconvulsive shock on the function, circuitry, and transcriptome of dentate gyrus granule neurons

**DOI:** 10.1038/s41386-026-02345-x

**Published:** 2026-02-04

**Authors:** Adrienne N. Santiago, Julia Castello Saval, Phi Nguyen, Hannah M. Chung, Shaharia Khan, Victor M. Luna, Rene Hen, Wei-li Chang

**Affiliations:** 1 Department of Psychiatry, Columbia University Irving Medical Center, New York, NY USA; 2 Division of Systems Neuroscience, New York State Psychiatric Institute, New York, NY USA; 3 Alzheimer’s Center at Temple, Department of Neural Sciences, Lewis Katz School of Medicine, Temple University, Philadelphia, PA USA

**Keywords:** Experimental models of disease, Stress and resilience, Depression, Adult neurogenesis, Limbic system

## Abstract

Therapeutic use of electroconvulsive shock (ECS) is particularly effective for treatment-resistant depression. Like other more common forms of antidepressant treatment, such as SSRIs, ECS has been shown to increase neurogenesis in the hippocampal dentate gyrus of rodent models. Yet the question of how ECS-induced neurogenesis supports improvement of depressive symptoms remains unknown. Here, we show that ECS-induced neurogenesis is necessary to improve depressive-like behavior of mice exposed to chronic corticosterone (Cort). We then use slice electrophysiology to show that optogenetic stimulation of adult-born neurons produces a greater hyperpolarization in mature granule neurons after ECS vs Sham treatment. We identify that this hyperpolarization requires the activation of group II metabotropic glutamate receptors. Consistent with this finding, we observe reduced expression of the immediate early gene cFos in the granule cell layer of ECS vs Sham subjects. Using single-nucleus RNA sequencing, we reveal major transcriptomic shifts in granule neurons after treatment with ECS+Cort or fluoxetine+Cort vs. Cort alone. We identify a population of immature cells that has greater representation in both ECS+Cort and fluoxetine+Cort treated samples vs Cort alone. We also find global differences in ECS- vs fluoxetine-induced transcriptomic shifts. Together, these findings highlight a critical role for immature granule cells in the antidepressant action of ECS.

## Introduction

In the United States, 21 million adults representing 8.3% of the population suffered at least one major depressive episode in a single year (NIMH reports, 2021). Selective serotonin reuptake inhibitors such as fluoxetine (Flx) remain the first-line therapy for major depression with a response rate of ~60%, including ~30–40% for full remission [1, 2]. Roughly a third of patients remain treatment resistant [3]. Therapeutic use of electroconvulsive shock (ECS) is the gold standard for effectiveness and remission of treatment-resistant depression [4–7], but the mechanisms of these effects remain unclear.

Similar to Flx [8], ECS increases neurogenesis in the hippocampal dentate gyrus (DG) of rodent models. ECS promotes the activation and proliferation of quiescent neural stem cells [9, 10], survival and differentiation of immature adult-born granule cells (iGCs) [11], and dendritic complexity, spinogenesis and axonogenesis of iGCs [12–14]. Human subjects treated with ECS exhibit more cells expressing biomarkers of iGCs, including doublecortin (DCX) and STMN1 [15]. Neurogenesis is necessary for Flx-induced antidepressant-like and anxiolytic-like effects in a variety of rodent behavioral paradigms, including coat-grooming [8], novelty-suppressed feeding (NSF) [8], and pattern separation [16], but not necessary for Flx effects in the forced swim test (FST) or open field tests [17]. Likewise, iGCs have been shown to be necessary for some anxiolytic effects of ECS, as pharmacogenetic deletion of iGCs blocks ECS-induced reduction of latency to feed in the NSF [13]. Yet the questions of whether and how ECS-induced neurogenesis supports the improvement of depressive-like symptoms remain unanswered.

Several properties of iGCs distinguish them from mature granule cells (mGCs) and provide insight as to their unique role in the DG network. Our group [18, 19] and others [20, 21] have shown that iGCs are more active than mGCs, yet paradoxically contribute to overall DG inhibition and maintenance of sparse network activity hypothesized to be necessary for optimal execution of DG-dependent behaviors [21, 22]. iGCs indirectly suppress mGC activity via inhibitory interneurons [19, 23–26] and directly inhibit mGCs via group II metabotropic glutamate receptor (mGluRII)-mediated activation of G-protein coupled inwardly rectifying potassium channels [27]. Delivery of systemic mGluRII agonist also reduces expression of the immediate early gene (IEG) cFos in the DG [28]. Here, we ask whether ECS likewise reduces mGC activity and test the role of mGluRII activity.

ECS and Flx both increase neurogenesis and produce antidepressant effects, yet ECS can effectively treat depression even after Flx has failed. We therefore hypothesize both converging and diverging mechanisms of action. To investigate this broadly, we used single-nucleus RNA sequencing (sn-RNAseq) to profile cell-type-specific transcriptomic changes in response to ECS or, as a comparator, Flx. We show here that ECS and Flx not only increase the population of iGCs in keeping with previous reports [12, 13, 29], but also result in distinct transcriptional changes in mGCs.

## Methods

### Mouse model

**Fig. 1:**
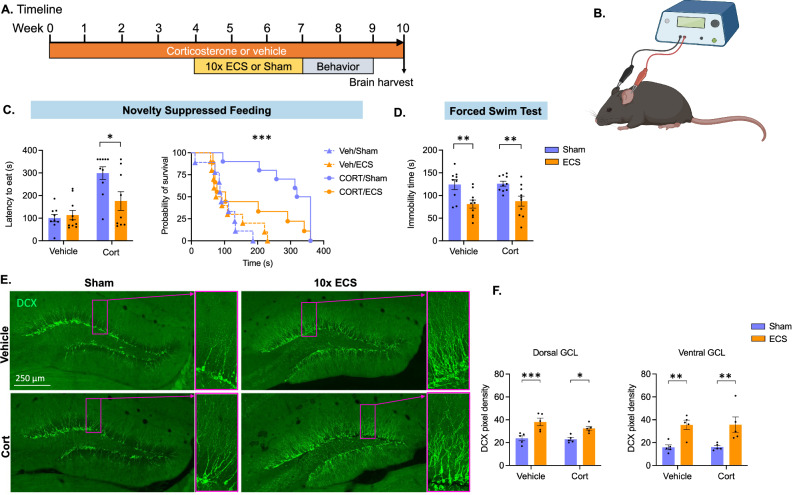
Mouse model of ECS. **A** Timeline of the experiment. 10-week-old mice were treated with Vehicle or ~5 mg/kg corticosterone (Cort) in drinking water to maintain stress throughout the experiment. After 4 weeks, mice received ECS or Sham treatment every other day for a total of 10 sessions and were tested in paradigms of anxiety-like and depression-like behavior starting 24 h after the last ECS session. Brains were harvested 1 week after the last behavioral test. **B** Electroshocks were delivered under isoflurane anesthesia by a Ugo-Basile ECS unit through ear-clipped electrodes at 120 Hz, 50 mA for 1 s, while Sham mice received anesthesia and ear clip placement, but no electroshock. **C** In the novelty suppressed feeding test, ECS-treated animals exhibit decreased latency to feed in the Cort group but not in the vehicle group. **D** In the forced swim test, time spent immobile is reduced after ECS both in the vehicle and the Cort groups. **E**, **F** Expression of the immature cell marker doublecortin (DCX) is greater in ECS vs Sham subjects. Statistical analysis was performed using two-way ANOVA for normally distributed data (**D**, **F**), and the log-rank Mantel-Cox test to report latency to feed (**C**); (* *p* ≤ 0.05, ** *p* ≤ 0.01, *** *p* ≤ 0.001).

All procedures were conducted in accordance with the USA NIH Guide for the Care and Use of Laboratory Animals and the Institutional Animal Care and Use Committees of New York State Psychiatric Institute and Columbia University. Mice were briefly anesthetized with 2% isoflurane in 1 L/min oxygen. Electroshocks were delivered to anesthetized mice through ear-clipped electrodes at 120 Hz and 50 mA, for 1 s (Fig. 1B). Ringer's salt solution was applied to the ear prior to shock to improve conductance. Mice received ECS every other day for a total of 10 sessions. Seizure was successfully induced in all ECS mice with no evidence of harm from the ECS procedure. Sham controls received isoflurane anesthesia and application of Ringer's solution and ear clips, with no delivery of electroshock current.

### Drug administration

As previously published [17, 30], Cort (Sigma) was dissolved in 0.45% betacyclodextrin (vehicle) and delivered via drinking water in opaque bottles, replaced every 3–4 days. Mice received 35 μg/ml of Cort, equivalent to ~5 mg/kg/day. Due to sex differences in response to chronic Cort stress [17, 31, 32], we limited this study to male mice.

### Behavior, IHC, X-IR, flow cytometry, and sn-RNAseq

Please see Supplementary Material.

### Statistical analysis

All statistical analyses were performed using Prism (GraphPad). Normally distributed data were analyzed by two-way ANOVA to assess ECS effects alongside experimental manipulations. Because NSF recordings were capped at 360 s, we frequently observed latency to feed measures that were censored at this limit. We therefore used pair-wise Log-rank Mantel-Cox tests for all NSF statistics and represented data with traditional bar graphs as well as a survival curve to show the impact of censoring on the data.

## Results

### Mouse model of ECS

10-week-old C57/Bl6 mice were treated with vehicle or ~5 mg/kg corticosterone (Cort) in drinking water to maintain a stress-like state throughout the experiment, (protocol as published [17]; Fig. 1A). After 4 weeks, mice from Vehicle and Cort groups underwent ECS or Sham treatment (Fig. 1B). Applied current induced tonic/clonic seizures, identifiable by acute arching of the back followed by rhythmic movement of limbs and sustained tension in the tail. Because repeated ECS is more effective than a single dose and more accurately mimics human treatment schedules [33–37], we repeated ECS or sham on alternate days for 10 sessions. Duration of seizure was unaffected by Cort vs vehicle administration (two-way ANOVA; F(1,9) = 0.07; *p* = 0.79; Fig. S1a).

We first established replication of published behavioral effects of ECS on anxiety-like and depression-like behaviors [13, 33]. During acclimation to behavioral testing, locomotion was observed in a simple arena for 10 min. Total locomotion did not differ significantly between groups (one-way ANOVA: *F* (3, 35) = 1.378; *p* = 0.2655; Fig. S1b). For the NSF, mice were deprived of food for 16 h before they were given access to a food pellet in the center of a novel arena. Latency to consume food is recorded for up to 360 s, before the test is repeated in the home cage. Latency to approach food specifically in the novel arena is an established indicator of anxiety-like behavior [8, 38]. In the novel arena, ECS-treated mice exhibited a decreased latency to feed in the Cort group (*p* = 0.0270) that was absent in the vehicle group (*p* = 0.6790, Log-rank Mantel-Cox test; Fig. 1C). By contrast, ECS had no effect on latency to feed observed within the home cage (two-way ANOVA *F* (1, 34) = 0.02409; *p* = 0.8776). In the FST, ECS mice spent less time immobile vs Sham in both the Cort (*p* = 0.0107) and Vehicle (*p* = 0.0052) treated groups (two-way ANOVA: *F* (1, 35) = 19.31; *p* < 0.0001; Sidak’s test post hoc; Fig. 1D). Together, these findings support previous publications establishing the anxiolytic and antidepressant-like effects of repeated ECS, particularly after exposure to chronic stress [13, 33].

### Effects of ECS on iGCs

A subset of 5 mice per group were analyzed for DCX expression (Fig. 1E). Estimating the relative density of DCX expression in the granule cell layer averaged over the full dorsoventral axis of the DG, we found that ECS treated mice had more DCX expression vs Sham mice, regardless of Cort (*p* = 0.0045) vs Vehicle (*p* = 0.0005) treatment (Fig. 1F; two-way ANOVA: *F* (1, 16) = 34.57; *p* < 0.0001; Sidak’s test post hoc). In dorsal DG, ECS-treated mice expressed a higher density of DCX vs Sham in both Cort (*p* = 0.0185) and vehicle-treated mice (*p* = 0.0008; two-way ANOVA: *F* (1, 16) = 27.56; *p* < 0.0001; Sidak’s test post hoc). In ventral DG, we again found that ECS-treated mice expressed a higher density of DCX vs Sham in both Cort (*p* = 0.0084) and vehicle-treated mice (*p* = 0.0080; two-way ANOVA: *F* (1, 16) = 22.38; Sidak’s test post hoc). Increased DCX expression was observed after 10X ECS, but not after a single dose of ECS (Fig. S2a). These findings are consistent with past publications that have reported increased neurogenesis after ECS as well as longer and more complex dendritic branches in DCX-immunoreactive dendrites [11, 14].

### Effects of X-irradiation to ablate DG neurogenesis on ECS efficacy

**Fig. 2:**
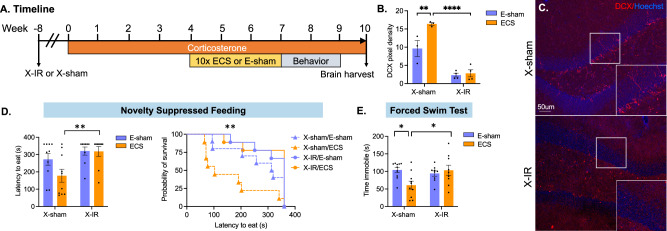
iGCs are required for the behavioral effects of ECS. **A** Experimental timeline. Mice received X-irradiation targeting the hippocampus (X-IR) or sham (X-sham). After 8 weeks of recovery, all mice received Cort in drinking water for the duration of the experiment. After 4 weeks, mice received 10 sessions of ECS or Sham (E-sham) and were behaviorally tested in the novelty suppressed feeding test and the forced swim test. **B**, **C** Doublecortin (DCX) immunohistochemistry reveals successful ablation of iGCs in X-IR mice vs. X-sham. **D** On the NSF test, latency to feed is reduced in ECS-treated mice with intact neurogenesis but not in X-IR mice treated with ECS. **E** On the FST, X-IR mice receiving ECS do not show decreased immobility time when compared to Sham-treated mice. Statistical analysis was performed using two-way ANOVA for normally distributed data (**D**), and the log-rank Mantel-Cox test to report latency to feed (**C**); (* *p* ≤ 0.05, ** *p *≤ 0.01, *** *p* ≤ 0.001).

To assess the role of iGCs in mediating the effects of ECS, we performed focal DG X-irradiation (X-IR) vs Sham (X-sham) followed by ECS vs Sham (E-sham) in Cort-treated mice (Fig. 2A). X-IR dramatically reduced expression of DCX (Fig. 2B, C), as shown previously [8, 27, 39, 40].

In the NSF test, we observed that ECS-treated mice had a higher latency to eat after X-IR vs ECS/X-sham mice (*p* = 0.0023, log-rank Mantel-Cox test; Fig. 2D). By contrast, X-IR had no significant impact on latency to eat within the home cage for either ECS (*p* =  0.3500) or E-sham groups (*p* = 0.254).

In the FST, ECS mice were immobile for significantly less time in the X-sham group vs X-IR-treated mice (*p* = 0.0155, two-way ANOVA interaction effect: *F* (1, 34) = 6.135; *p* < 0.0184; Sidak’s test post hoc; Fig. 2E). Within the X-sham group, ECS mice spent less time immobile vs E-sham mice (*p* = 0.0107). Together, these findings support a critical role for iGCs in mediating ECS-induced improvement of both anxiety-like and depressive-like behaviors.

### Effects of 10x ECS on iGC-driven inhibition of mGCs

**Fig. 3:**
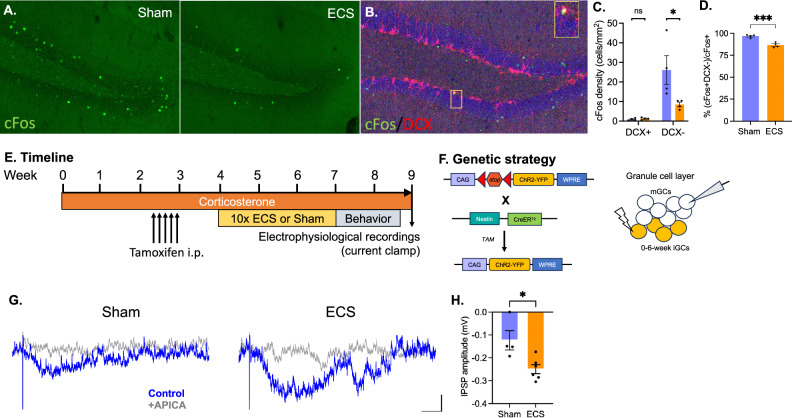
mGluRII mediates iGC-driven inhibition in mGCs after 10x ECS. **A**, **B** cFos expression in the granule cell layer of the dentate gyrus following rest in the home cage is lower in mice that received 10x ECS vs Sham controls, suggesting that ECS supports a sparser network at rest. **C** Image of a single confocal plane reveals co-localization of cFos in a DCX+ cell (inset) in the DG of an ECS-treated mouse. DCX was used to identify whether cFos-expressing cells were iGCs (DCX-positive) vs mGCs (DCX-negative). **D** Within the cFos-expressing population, ECS significantly reduced the proportion of DCX-negative mGCs active at rest in the homecage. **E** Experimental timeline. Mice received Cort in drinking water for 4 weeks. Six weeks after tamoxifen injection to induce expression of ChR2-EYFP in iGCs, whole-cell recordings were performed in mature granule cells in DG brain slices. Delivery of 10x ECS or Sham occurred one week after tamoxifen-induced Cre recombination. **F** Breeding strategy and experimental protocol for electrophysiological approach. Nestin-CreER^T2^ mice were crossed with floxed Channelrhodopsin-EYFP (ChR2-EYFP) expressing mice. Optogenetic stimulation of iGCs evoked responses in mGCs. **G** We optogenetically stimulated 0-6-week-old iGCs to evoke inhibitory postsynaptic potential in mGCs in the presence of 20 mM NBQX, 50 mM APV, and 20 mM bicuculline (blue trace). These inhibitory signals were blocked by bath application of the mGluRII antagonist APICA (500 mM; gray trace; scale bar = 200 ms, 100 mV). **H** mGluRII-mediated IPSP had greater negative amplitude after ECS vs Sham in Cort-treated mice. (* *p *≤ 0.05, *** *p* ≤ 0.001).

Several studies from our group [19, 22, 27] and others [20, 21] have suggested that iGCs support DG function by decreasing activity in mGCs. To assess whether DG activity was sparser after ECS vs Sham, we quantified cFos expression after rest in the home cage (Fig. 3A). We found that cFos-positive cell density was lower in ECS vs Sham-treated mice (Student’s T test; *p* = 0.0007). After 1 ECS session, cFos density was reduced, but this effect was not sustained at 21 days; in contrast, after 10 ECS sessions, cFos cell density was reduced, and this effect was sustained in keeping with the fact that neurogenesis increases after 10 sessions of ECS but not after one (Fig. S2b). cFos-expressing cells were most frequently found in the outer granule cell layer, farthest from the neurogenic subgranular zone, suggesting a likelihood that active cells are predominantly mGCs [41]. To verify this, we double-labeled tissue with both cFos and DCX and analyzed a single confocal plane per section to confirm potential antibody colocalization (Fig. 3B). While we found no treatment effect on DCX+cFos+ cell density (*p* = 0.9973; sidak test post-hoc), we did observe a significant decrease specifically in the density of cFos+ DCX-negative mGCs in ECS-treated subjects (*p* = 0.0119, sidak test posthoc; ANOVA for treatment effect *F* (1, 12) = 5.341, *p* = 0.0394; Fig. 3C). We found that most cFos+ cells are DCX-negative mGCs in both sham (96.69%) and ECS (86.63%) groups, and that the percentage of cFos+ cells identified as DCX-negative mGCs is lower in ECS vs Sham subjects (Student’s *t*-test; *p* = 0.0010; Fig. 3D).

iGCs are capable of directly inhibiting mGCs via mGluRII-mediated postsynaptic hyperpolarization [27]. To determine whether this type of inhibition was enhanced by ECS vs Sham in subjects maintained on Cort, we used whole-cell current clamp recordings in DG brain slices (Fig. 3E). For these experiments, we crossed a Nestin-CreER^T2^ mouse line with a floxed channelrhodopsin-EYFP (ChR2-EYFP) expressing mouse line, which enabled us to inducibly express ChR2-EYFP in iGCs upon delivery of tamoxifen (Fig. 3F). We optogenetically stimulated 0- to 6-week-old iGCs and recorded synaptic responses from mGCs in the presence of AMPA-, NMDA-, and GABAA-receptor antagonists [27] (Fig. 3G). We observed significantly larger inhibitory response in mGCs of ECS-treated mice than Sham controls (Fig. 3F; Sham = –0.12 ± 0.04 mV, *N* = 4; ECS = 0.25 ± 0.02, *N* = 6; Student T-test, *p* = 0.0162). The inhibitory currents were blocked with bath application of the mGluRII antagonist, APICA, indicating that direct iGC hyperpolarization of mGCs requires mGluRII, as previously shown [27]. Together, these findings suggest that iGC activation may contribute to the sparsity of the granule cell network by engaging mGluRII present on mGCs.

### Both ECS and Flx drive transcriptomic shifts, indicating greater neuroplasticity and a more prominent immature phenotype among granule neurons

**Fig. 4:**
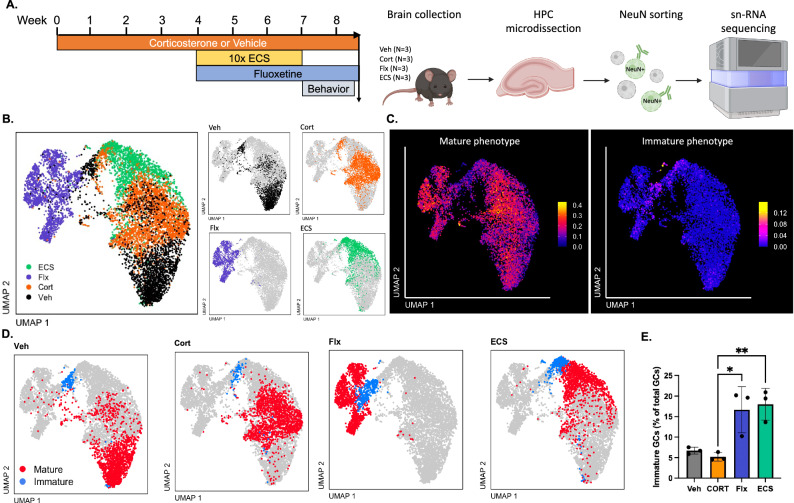
Both ECS and Fluoxetine drive transcriptomic shifts, indicating greater neuroplasticity and a more prominent immature phenotype among granule neurons. **A** Single-nucleus RNA sequencing was performed on hippocampal nuclei from four treatment groups: vehicle (Veh), corticosterone (Cort), Cort plus fluoxetine (Flx), and Cort plus 10x ECS (*N* = 3 per group). Neuronal nuclei were identified with the NeuN antibody, followed by cell sorting. UMAP clustering of all neurons revealed a granule neuron population that was used for all subsequent analyses. **B** UMAP projections of the granule neuron population, labeled by treatment condition. **C** Genes identified in Hochgerner et al 2018 as those predominantly expressed in either mature or immature cell types were used to produce mature vs immature phenotype module scores. UMAP projections depict granule neurons from all treatment conditions, while color encodes the intensity of expression of mature vs immature module scores. **D** Mature vs immature phenotype module score was used to identify mature vs immature cell clusters. UMAP clustering of mature (red) and immature (blue) nuclei by treatment condition is depicted. **E** The percent of granule neurons in the immature cell cluster is greater for Flx and ECS-treated mice vs Cort alone. Each point represents a single mouse (One-way ANOVA with Tukey post hoc, * *p* ≤ 0.05, ** *p* ≤ 0.01).

To characterize granule neuron response to antidepressant treatment, we performed sn-RNAseq on dissected hippocampi from four treatment groups: vehicle (Veh), Cort, Cort plus Flx, and Cort plus 10x ECS (*N* = 3 per group; Fig. 4A). Neuronal nuclei were isolated using fluorescent activated cell sorting for NeuN+ nuclei. Transcriptomic profiles of hippocampal neuronal nuclei were generated, and granule cells were identified by expression of *Prox1*, *Dock10*, and *Stxbp6* (*N* = 8318 nuclei). We then used Uniform Manifold Approximation and Projection (UMAP) to reduce the dimensionality of granule neuron RNA profiles to two axes to visualize relative similarity vs difference in expression profile between individual neurons (Fig. 4B). We observed that nuclei from Veh, Cort, Flx, and ECS groups clustered more tightly within vs across groups, indicating that granule neurons from each group are transcriptomically distinct (Fig. 4B). If Flx or ECS simply reversed the effect of Cort, we may expect to see more overlap between these groups and the vehicle group, yet this was not the case.

To further investigate the role of iGCs, we used differentially expressed genes (DEGs) identified by Hochgerner et al. [42] as characteristic of iGCs vs mGCs to create module scores of immature vs mature phenotype (Fig. 4C). We found that, in our sample, immature vs mature phenotype cells produced distinct clusters (Fig. 4D). Interestingly, the percent of neurons in the immature phenotype cluster differed by treatment group (Fig. 4E; one-way ANOVA, F (3, 8) = 11.00, *p* = 0.0044). Post hoc Tukey’s tests revealed that both Flx (*p *= 0.0152) and ECS groups (*p* = 0.0083) had a significantly greater proportion of immature-clustered granule cells vs Cort.

Both ECS and Flx groups exhibited upregulation (vs Cort) of key genes known to play a critical role in neurodevelopment and neuroplasticity (see Supplementary Table 1 for a full list of DEGs for ECS vs Cort and Flx vs Cort). These include Neuregulin 1 and 3 (*Nrg1*, *Nrg3*), Neurexin 3 (*Nrxn3*), and *Ncam2*. ECS also upregulates *Nrxn1*, while Flx upregulates both *Bdnf* and the gene encoding TrkB (*Ntrk2*). Both ECS and Flx also exhibit downregulation of genes associated with mGC identity [43], including *Calb1*, *Dock10*, Camk2a, and *Rbfox3*.

**Fig. 5:**
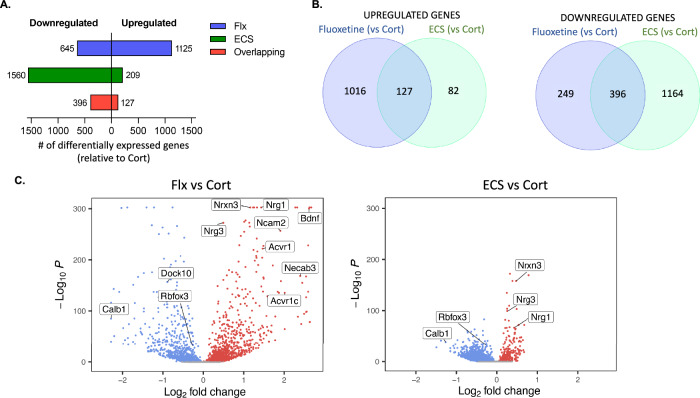
Treatment with fluoxetine induces greater transcriptome upregulation while ECS induces a downward transcriptomic shift. **A** Representation of the number of differentially expressed genes (DEGs) that are upregulated (right) vs downregulated (left) for Flx and ECS groups, relative to Cort expression. **B** Venn diagram depicting overlap of upregulated vs downregulated DEGs in Flx and ECS groups, vs Cort. **C** Volcano plot depicting expression level (-Log of the p value by Log2 fold change) of DEGs for Flx and ECS groups, vs Cort.

Consistent with our UMAP results (Fig. 4B), we find big differences in Flx- and ECS-induced transcriptomic shifts. While Flx induces upregulation of 1125 DEGs and downregulation of 645 DEGs, ECS upregulates only 209 DEGs and downregulates 1560 DEGs (Fig. 5A, B). Flx also induces a stronger transcriptomic response, with greater average fold change and -LogP value for DEGs (Fig. 5C, D). Thus, ECS achieves equivalent behavioral antidepressant effects vs Flx while unexpectedly inducing a more modest transcriptomic shift. Interestingly, this pattern is also observed in CA1 and CA3 cells (Fig. S4). For CA1, Flx induces upregulation of 598 genes and downregulation of 258 genes, while ECS induces upregulation of 94 genes and downregulation of 354 genes. For CA3, Flx induces upregulation of 212 genes and downregulation of 85 genes, while ECS induces upregulation of 33 genes and downregulation of 49 genes. We also observed antidepressant upregulation of Nrg3, Nrxn3, and Ncam2, as well as downregulation of Camk2a in both CA1 and CA3, which was also observed in granule neurons. Calb1 was also downregulated in both granule neurons as well as in CA1and CA3, and Calb1 downregulation has been proposed to correspond to a dematuration of the DG [44, 45].

## Discussion

### Summary of results

Here, we demonstrated that ECS-induced anxiolytic- and antidepressant-like effects require iGCs. We show for the first time that ECS amplifies direct iGC-induced hyperpolarization of mGCs via mGluRII. We then used sn-RNAseq to characterize the transcriptomic shift induced by ECS or Flx vs Cort alone. We show that ECS and Flx both induce a shift towards greater expression of an immature vs mature granule neuron profile, but that the overall expression patterns of the two interventions are distinct.

### iGCs and the sparse DG

Our behavioral, histochemical, electrophysiological, and transcriptomic data converge in supporting the hypothesis that, despite comprising a relatively small population of cells, iGCs play a critical role in the antidepressant action of ECS. While previous publications have detailed the neurogenic effects of ECS [9, 11], this is the first study to demonstrate that ECS facilitates iGC-driven inhibition of mGCs. We also demonstrate that ECS reduces cFos expression specifically in mGCs weeks after seizure cessation, which parallels previous work using whole hippocampus RT-PCR to show that ECS decreases long-term expression of the IEGs *Arc*, *Fos*, and *Egr1* [46, 47].

Optogenetic activation of iGCs has been shown to increase the sparsity of mGC calcium transients, while optogenetic inhibition of iGCs decreases mGC sparsity [21]. Likewise, genetic manipulation to increase neurogenesis results in decreased DG activity, while X-IR neurogenesis ablation increases DG activity [24]. One recent publication found that X-IR did not significantly reduce mGC activity in cue or place cells during contextual discrimination, but instead impaired mGC contextual remapping [40]. This finding suggests that iGCs may have task-dependent effects on mGC activity, which may impact the control iGCs have over the inhibitory tone of the DG. We hypothesize that iGC-induced suppression of mGCs contributes to the sparse DG network activity shown to support pattern separation and stress-resilience [22].

Our past work has shown that iGCs can directly inhibit mGCs via mGluRII-mediated activation of G protein-coupled inwardly rectifying potassium currents (GIRKs) [27]. Group II mGluRs include both mGluR2 and mGluR3, but only mGluR2 is known to induce hyperpolarization via GIRKs, which co-express on mGC proximal dendrites [48]. Interestingly, expression of both mGluR2 and GIRKs is absent in iGCs and increases with maturity [49, 50]. This suggests that the electrophysiological effects of mGluR2 that we observe are specific to the mGC population. Likewise, the antidepressant-like effects of Flx are mediated by 5-HT1A receptors (an inhibitory GPCR like mGluR2) also expressed on mGCs [51]. Directly inhibiting mGCs with DREADDs also produces a stress-resilient phenotype [22]. Together, these results suggest that mGC inhibition is a key component in antidepressant response and stress resilience [22, 51].

### The potential role of granule neurons in mediating therapeutic vs side effects of ECS

In addition to improving symptoms of depression, ECS therapy is also known to produce mild to severe retrograde amnesia [52], which has been observed in rodent models of ECS [53]. Due to its well-known role in memory formation, the hippocampus is a focal point for research on ECS-induced amnesia [46, 54, 55]. Our finding that DG neurogenesis is necessary for the antidepressant-like effects of ECS suggests that the DG should be investigated in parallel for its potential contribution to therapeutic effects. This finding is supported by a previous report using genetic techniques to ablate neurogenesis, which likewise reduced ECS-induced anxiolytic-like effects [13]. We also show that ECS increased neurogenesis over the full dorsoventral axis of the DG. While ECS-induced neurogenesis in the ventral DG may contribute to antidepressant-like effects, as we have shown in the case of Flx [39], it is possible that neurogenesis in the dorsal DG may contribute to ECS-induced amnesia [54].

We found that chronic Cort administration produced depressive-like behavior, which was rescued by ECS (Fig. 1). For this reason, our transcriptomic data compares ECS with Cort to Cort alone. This parallels use in patient populations, where ECS is administered in cases of severe depressive behavior. However, the Cort model for chronic stress is more effective in male than female mice [17, 31, 32]. We therefore limited our study to males. Future studies using a different model for chronic stress will benefit from the inclusion of female subjects.

### ECS and Flx increase transcription factors associated with neuroplasticity and immaturity

Since ECS and Flx both produce antidepressant-like effects and increase neurogenesis, we hypothesized that both manipulations would produce some similar transcriptomic changes in the DG. Indeed, we found that both Flx and ECS upregulated genes critical for iGC survival, growth, migration and connectivity, including the neurexins [56], neuregulins [57], and *Ncam2* [58]. We then classified granule neurons into immature- vs mature-phenotype clusters, and found a greater number of immature-phenotype cells in the Flx- and ECS-treated populations. We used NeuN sorting to enrich for neurons in our sn-RNAseq data, which excludes the most immature cell types (e.g., neural stem cells, neuroblasts). Our results show that *Rbfox3*, which encodes NeuN, is downregulated in both ECS and Flx groups. Despite potentially losing a greater number of early iGCs that express little NeuN, antidepressant-treated groups nonetheless had more immature-phenotype cells vs controls. This finding parallels work using BrdU and DCX labeling, in situ hybridization, and neuronal tracing to show that both Flx [17] and ECS [9, 11] promote the survival, growth, and dendritic arborization of adult-born iGCs.

One of the advantages of sn-RNAseq is the ability to profile maturity vs immaturity according to a broad array of gene expression patterns [42, 59], rather than relying solely on a few canonical markers, such as DCX. This allows us to identify a larger, more diverse population of cells with an immature expression profile [59]. In addition to stimulating neurogenesis, ECS and Flx may also prolong the immature state of iGCs by downregulating expression of factors associated with late-stage iGC maturation [11], as our sn-RNAseq results suggest (Figs. 4 and 5). Additionally, ECS and Flx may induce an immature phenotype among embryonic- and adult-born mGCs that are outside the experimentally-defined 4–8-week window of immaturity [43, 44]. While this dematuration-like phenomenon may contribute to the DCX-negative immature-phenotype population, we recently demonstrated that the expression of DCX in granule cells is exclusively driven by adult-born iGCs [60]. Therefore, it is unlikely that the observed increase in DCX is due to dematuration. Collectively, our data suggest that Flx and ECS increase neurogenesis as well as the number of cells in the transitional space between immature and mature phenotypes, which could be facilitated by either increased dematuration of mGCs or slower maturation of iGCs.

### Converging and diverging mechanisms in ECS and Flx treatments

We also identified key differences in granule cell transcription profiles after Flx and ECS. We observed that Flx produces a greater transcriptomic shift dominated by gene upregulation, while ECS produces a more modest transcriptomic shift dominated by gene downregulation. Experimental timing may contribute to these differences, as Flx was administered continuously until tissue harvest, while ECS mice were sacrificed 1.5 weeks after their final session. Indeed, transcriptomic effects of ECS ~90 min post-seizure are notably robust and positive, particularly among IEGs [61, 62], as one might expect. Our timepoint for brain harvest was selected to align with confirmed antidepressant-like behavior (Fig. 1) and parallel the timeline of observed efficacy of Flx vs ECS in patients: ECS therapy can have sustained antidepressant effects for months and even years following treatment, while the efficacy of Flx often lowers measurably when patients stop treatment [36, 37, 63]. Alternatively, some of the effects of Flx on its many downstream serotonin receptors may have little to do with antidepressant effects [64].

There are several additional notable differences in ECS vs Flx transcription profiles. Interestingly, Flx, but not ECS, upregulates both *Bdnf* and the gene encoding its primary receptor, TrkB, which are known to promote neurogenesis and mediate antidepressant-like effects [65]. This indicates that Flx and ECS may engage different mechanisms to increase neurogenesis and promote antidepressant-like effects. While we have shown here that the relatively small population of iGCs is necessary for the antidepressant-like effects of ECS, simply amplifying the iGC number is not sufficient to produce all antidepressant-like effects [16, 17, 66]. We hypothesize that, while iGCs are a necessary component of the antidepressant effects of both ECS and Flx, additional mGC responses are likely to contribute and may also be required to produce antidepressant-like behaviors [51]. Our diverging transcriptomic data reveal that these factors may be different for ECS vs Flx therapies, which may reflect differences in therapeutic responses and side effects reported in clinical applications.

## Conclusions

Overall, these findings provide insight into the neurobiological mechanisms underlying the antidepressant effects of ECS. We found that ECS-induced stress resilience requires adult hippocampal neurogenesis, and that ECS amplifies iGC-induced hyperpolarization of mGCs via mGluRII. We also found that ECS and Flx both induce a transcriptomic shift towards a more immature phenotype in granule neurons; however, ECS and Flx elicit distinct transcriptomic changes in mature DG neurons. This likely contributes to their different antidepressant profiles in clinical application, including differences in treatment effectiveness, effect onset and duration, as well as differences in side-effects, like memory loss.

## Supplementary information

Supplementary material

Supplementary data tables

## Data Availability

Please find a complete list of DEGs for ECS vs Cort and Flx vs Cort datasets, as well as a list of overlapping vs non-overlapping DEGs in the Supplementary Tables. Original data and scripts for sn-RNAseq data are all publicly available on our lab’s github website:https://github.com/pnguyen1003/snRNA-seq-analyses
