# Supplementary material for: Effects of electroconvulsive shock on the function, circuitry, and transcriptome of dentate gyrus granule neurons
Source: Neuropsychopharmacology. 2026 Feb 4;51(7):1258–66. doi: 10.1038/s41386-026-02345-x (PMC13212720; doi:10.1038/s41386-026-02345-x)
Supplement: Supplementary file 1 — Supplementary material [file 41386_2026_2345_MOESM1_ESM.docx]

**Supplemental methods and figures.**

**Supplemental methods.**

**Mice**.

All procedures were conducted in accordance with the U.S. NIH Guide for the Care and Use of Laboratory Animals and the Institutional Animal Care and Use Committees of New York State Psychiatric Institute and Columbia University. Adult male mice were housed in a vivarium grouped 2-4 mice/cage, maintained on a 12-hour light cycle. For all experiments, mice began experimental procedures at 10 weeks of age. C57/6J mice were obtained from Jackson Laboratory. Nestin-CreER^T2^ transgenic mice [65] were bred in house on a C57/6J background (Fig 3). Schematic diagrams illustrating ECS (Fig 1b) and sn-RNAseq (Fig 4a) methods were created using BioRender (2024). To induce ECS, electroshocks were delivered to anesthetized mice through ear clipped electrodes at 120 Hz and 50 mA, for a duration of 1 second (Fig 1b). This current induced tonic/clonic seizures, identifiable by acute arching of the back followed by rhythmic movement of limbs and sustained tension in the tail.

**IHC.** For histology, mice were transcardially perfused with 4% paraformaldehyde (PFA; EMS) in 1X phosphate buffer solution (PBS), after which brains were removed and post-fixed in 4% PFA for 24 h. They were transferred to a 30% sucrose solution in 1X PBS for 2 days, after which they were flash-frozen in methylbutane and coronally sliced on a cryostat (Leica CM 3050S) at a thickness of 40 μm. Single immunohistochemistry for DCX and cFos were performed as previously described [19]. Briefly, for DCX, antigen retrieval was performed with heat treatment to 80C in pH8 citrate buffer. Tissue was then incubated in blocking buffer containing 10% normal donkey serum and 0.2% triton for 1 hr. Primary antibody (Cell Signaling Tech #46042S) was diluted 1:400 and incubated with tissue overnight at 4C. After washing 3X in PBS, Alexa Fluor donkey anti-rabbit 488 secondary antibody was used. For cFos, (Cell Signaling Tech #2250), antibody was diluted 1:400 and no antigen retrieval step was applied. For double labeling of cFos and DCX, we switched to a guinea pig DCX antibody (Synaptic Systems, #326014, diluted 1:500) with no antigen retrieval. All slides were imaged on a Leica SP8 and all IHC images were analyzed with ImageJ software (NIH), as previously reported [66].

**Focal X-irradiation.**

Hippocampal irradiation was conducted to eliminate DG neurogenesis, as previously described [8,27,38,39]. After anesthesia with 6 mg/kg sodium pentobarbital i.p., 10-week-old mice were placed in a stereotaxic frame and covered by a lead shield with a 3.22 x 11-mm window positioned to allow focal application of X-irradiation to the hippocampus. X-rays were filtered using a 2 mm Al filter, the corrected dose rate was approximately 1.8 Gy per min and the source to skin distance was 30 cm. A cumulative dose of 5 Gy was given over the course of 2 minutes and 47 seconds over 3 days, with 3 days between each X-ray session. Control mice did not receive radiation, but were anesthetized with irradiated mice throughout the experiment.

**Behavior**.

Novelty-suppressed feeding (NSF) was performed as previously described [37]. Briefly, mice are food deprived for 16 hrs before placement in a brightly lit arena with a food pellet secured to the center of the arena. This provides a conflict anxiety test whereby the motivation to eat competes with the risk to enter the center of a brightly lit arena. After this measure is recorded, mice are placed with a food pellet in their home cage, and latency to bite food is recorded. Forced-swim test (FST) was performed as previously described [64]. Briefly, 3L beakers were filled with 2500 ml of tap water at 25C. Time spent actively swimming vs passively floating was recorded using Videotrack software (ViewPoint Behavior Technologies).

**Electrophysiology**.

We performed whole-cell current clamp recordings on GCs while optogenetically manipulating immature iGCs as previously described [27].

**Flow cytometry**

For fluorescence activated nuclei sorting (FANS), flow cytometry experiments were performed as previously described [67]. Briefly, animals were perfused transcardially with ice-cold 1X PBS and intact hippocampi were isolated under a dissecting microscope where meninges and excess myelin were removed. Hippocampi (2 per mouse) were mechanically homogenized using a 2 mL glass tissue homogenizer (Kontes Glassware) on ice using twelve strokes of the tight pestle in 1% BSA-PBS (Sigma-Aldrich, A3059). Nuclei were filtered using a 40um strainer and then pelleted at 300 g for 5 minutes at 4°C. Nuclei were then resuspended in 22% Percoll (GE Healthcare) in PBS and centrifuged at 900 g for 20 minutes with acceleration set to 4 and deceleration set to 1 to remove cellular debris. Pelleted nuclei were then washed with PBS and incubated in block solution consisting of 1% BSA-PBS for 10 min prior to staining with mouse anti-NeuN antibody conjugated to Alexa Fluor 488 (EMD Millipore, MAB377X) at 1:1000 concentration for 10 min on ice. Cell hashing was performed with CellPlex Multiplexing Oligos (CMO; 10x Genomics, 3’ CellPlex Kit Set A, 1000261). CMOs were added to nuclei after primary antibody incubation and samples were incubated at room temperature for an additional 5 min. After 2 additional wash steps with PBS, biological replicates were pooled together prior to fluorescence activated nuclei sorting. All buffers contained Protector RNase Inhibitor (Sigma-Aldrich, 03335399001) at 0.2 U/uL for downstream sn-RNAseq experiments. DAPI (Sigma-Aldrich, D9542) was added to each sample immediately before sorting to identify nuclei. Nuclei were sorted on a Sony MA900 Cell Sorter and gated on forward/side scatter, DAPI, and NeuN. Flow cytometry and cell sorting experiments were performed in the Columbia Stem Cell Initiative Flow Cytometry core facility at Columbia University Irving Medical Center under the leadership of Dr. Michael Kissner. All data analysis was performed using FlowJo software.

**Single nuclei RNA-sequencing**

**Library preparation and sequencing**

FANS purified neuronal nuclei were sequenced using the 10 Genomics Single Cell Gene Expression 3’ platform (v3). Nuclei barcoded with cell multiplexing oligos were pooled into 4 samples and approximately 15,000 nuclei per sample were loaded into each well of Chromium Chip A and combined into droplets with barcoded beads using the Chromium controller according to the manufacturer instructions. Libraries were prepared by the JP Sulzberger Columbia Genome Center following the instructions in the Chromium Single Cell 3′ Reagent Kits version 3 user guide and sequenced using an Illumina Novaseq 6000 sequencer. Samples were sequenced to 45K-60K mean reads per cell with sequencing saturation of ≥33%.

Sequenced samples were processed using the Cell Ranger 6.1.2 pipeline and aligned to the mm10-2020-A mouse reference genome. Approximately 70% of all nuclei were successfully assigned to a CMO barcode using the ‘cellranger multi’ pipeline. Approximately 25,974 total nuclei were recovered prior to quality control processing.

**Quality control and data analysis**

Quality control, clustering, and differential expression analyses were conducted in R v4.3.0 using Seurat v4.4.0 38, 39.

Doublets were identified as nuclei with more than 1 unique CMO barcode, comprising ~5% of total nuclei, and were removed prior to downstream analyses. Cells with fewer than 1600 or over 3600 detected genes/cell, or more than 5% mitochondrial DNA were excluded prior to downstream analyses. Genes that were expressed by fewer than 5 cells in the dataset were also excluded. After filtering for these quality control metrics, we recovered 22,199 total nuclei (5919 vehicle, 6313 Cort, 5323 Cort + Fluoxetine, and 4644 Cort + ECS); see also supplemental figure S2.

Following alignment in Cell Ranger (as described above), molecular counts data were imported into R and analyzed using the Seurat package v4.4.0. Counts data underwent normalization and variance stabilization, regressing out percent mitochondrial RNA and total counts per cell, using the sctransform function in Seurat. The top 6000 most variable genes were used to calculate 50 principal components (PCs), and the top 35 PCs were used for nearest neighbor, UMAP, and clustering. Cell types were identified by expression of known cell type-specific markers [68]. Dentate granule cells were isolated using the pan-granule cell markers Prox1, Dock10, and Stxbp6.

Differential gene expression between treatment conditions were calculated using the MAST test in the FindMarkers function in Seurat. Only genes expressed in 25% of the cells in a given condition and a minimal log fold change threshold of 0.25 were included in the differentially expressed gene list. GO analysis was conducted using the Metascape webpage (https://www.metascape.org). Both upregulated and downregulated genes were included in bar plots.

Gene lists for mature or immature granule cell profiles were derived from Hochgerner et al., 2018. The defining features of mature vs immature granule cell populations from the Hochgerner et al., 2018 dataset were selected using available metadata provided by the authors in their online submission of the data. Their publicly available dataset defines key features of an immature cluster entitled “Immature-GC” and key features of a mature GC cluster, entitled “GC-adult” in their data. The “AddModuleScore” function in Seurat was used to generate module scores of mature and immature granule cell profiles.

Data and scripts used are all publicly available on our lab’s github website:

[https://github.com/pnguyen1003/snRNA-seq-analyses](https://nam02.safelinks.protection.outlook.com/?url=https%3A%2F%2Fgithub.com%2Fpnguyen1003%2FsnRNA-seq-analyses&data=05%7C02%7Cans2112%40cumc.columbia.edu%7C091e1a7be555464d4c9e08dd792db671%7Cb0002a9b0017404d97dc3d3bab09be81%7C0%7C0%7C638799960062130584%7CUnknown%7CTWFpbGZsb3d8eyJFbXB0eU1hcGkiOnRydWUsIlYiOiIwLjAuMDAwMCIsIlAiOiJXaW4zMiIsIkFOIjoiTWFpbCIsIldUIjoyfQ%3D%3D%7C0%7C%7C%7C&sdata=nHP4fP%2FxzXlVUEmstm7HU3CO3lCyweWc7UAzGD%2Bie5s%3D&reserved=0)

**Supplemental Figure 1.**

**
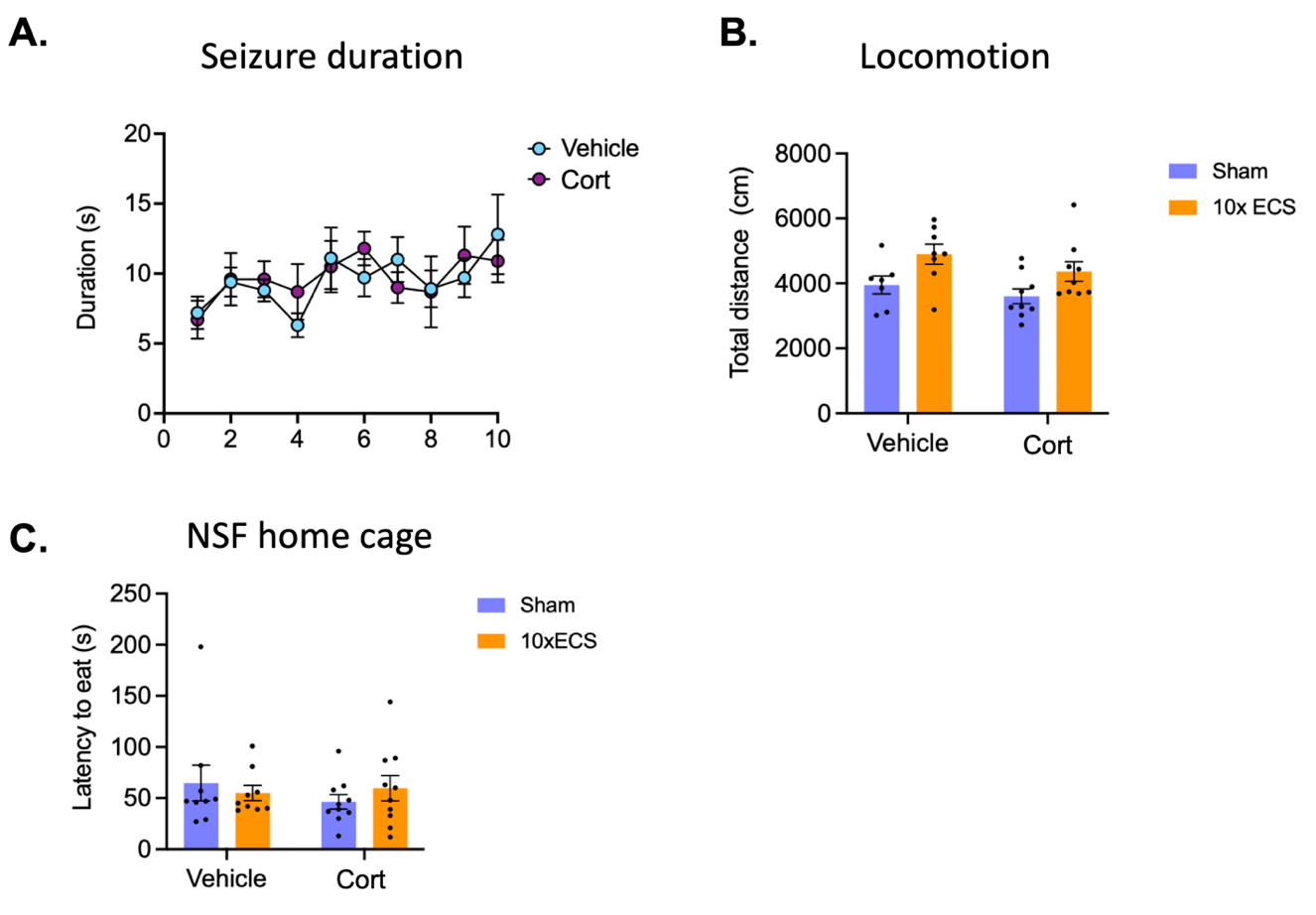
**

**S1.** **Supplemental figure 1. Additional characterization of the mouse model of ECS.** **A**. Treatment with corticosterone (Cort, purple) vs vehicle (blue) had no impact on duration of seizure. **B**. Locomotion was measured in an open arena. While two-way ANOVA revealed an effect of ECS treatment (F (1, 29) = 9.200; p = .0051), post hoc Sidak’s test revealed no significant differences in vehicle (p = .0601) or Cort treated subjects (p = .1054). **C**. For the NSF, no differences in latency to eat in the home cage were observed.

**S2. Supplemental figure 2.**


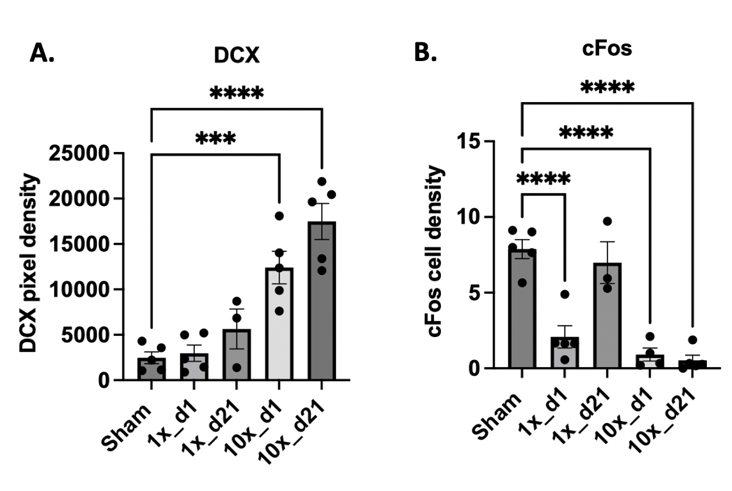


**S2. Supplemental Figure 2. Comparison of sham subjects to subjects with a single ECS treatment (1X) vs 10 ECS sessions, given every other day (10X ECS) and sacrificed either one day (1d) after the last ECS session, or 21 days (21d) after the last ECS session. A.** One-way ANOVA revealed significant effects on DCX expression (F (4, 18) = 19.20; p < .0001). We found no difference in DCX expression after 1X ECS, but significantly increased DCX expression after 10X ECS at both 1 day after the last ECS session (p = .0005) and 21 days after the last ECS session (p < .0001). **B.** One-way ANOVA revealed significant effects on cFos expression (F (4, 17) = 26.14, p < .0001). cFos expression was lower than sham 1 day after 1X ECS (p < .0001), with no effect of 1X ECS after 21 days. By contrast, 10X ECS was significantly lower both after 1 day (p < .0001) and after 21 days (p < .0001).

This finding supports the work of several previous publications which suggest a dose-dependent effect of ECS on cell proliferation and DCX expression (summarized in An and Wang’s 2022 review; PMID: 35063747). It is also consistent with our hypothesis that the decrease in c-fos expression after 10 doses of ECS is due to increased neurogenesis which leads to greater inhibition of mature granule cells.

**S3. Supplemental figure 3.**

**
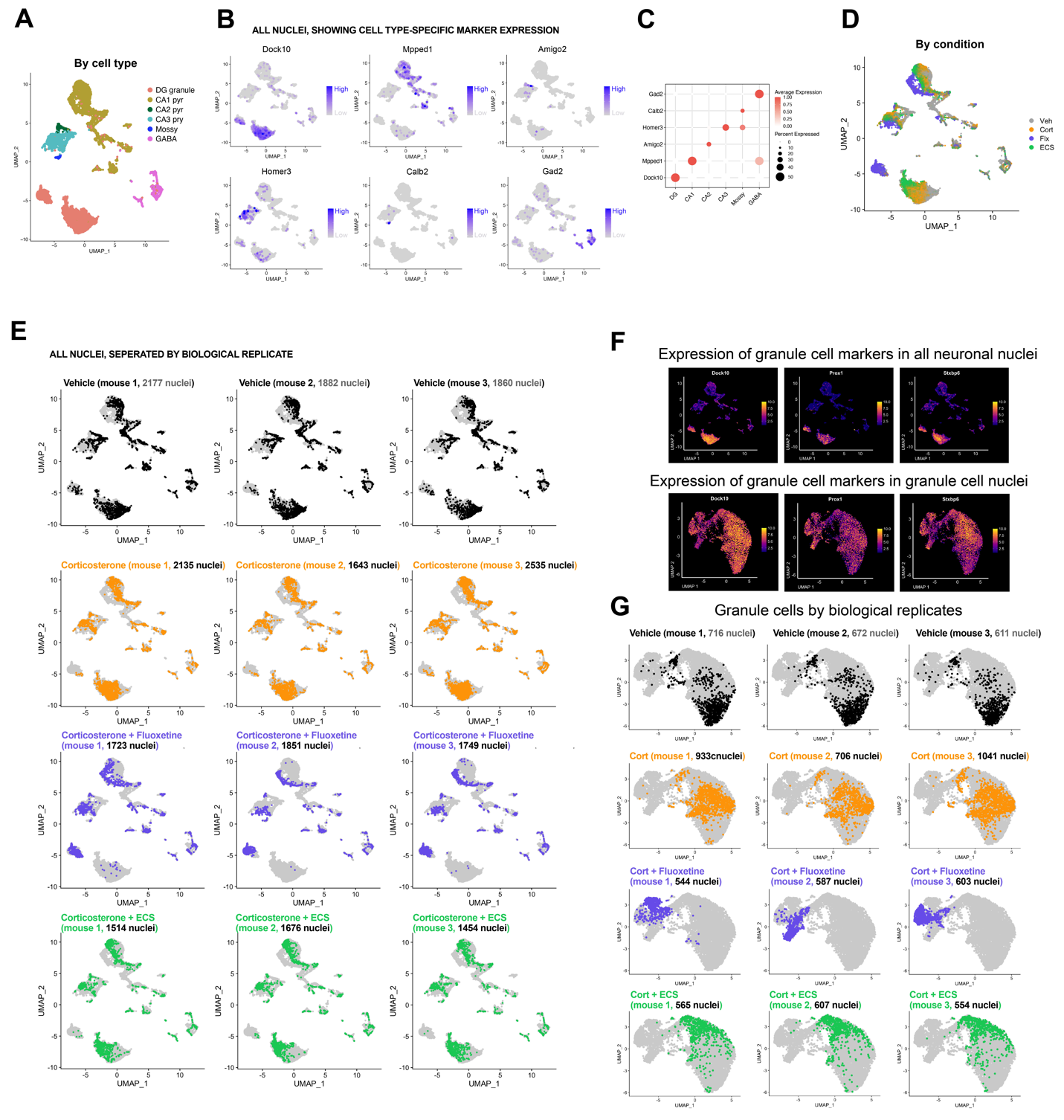
**

**S3.** **Supplemental figure 3.** **A-B.** Quality control metrics for single nuclei RNA-sequencing in vehicle, corticosterone, corticosterone + fluoxetine, and corticosterone + ECS groups. Dashed lines indicate minimum and maximum threshold settings. **A.** Unsupervised clustering (uniform manifold approximation and projection (UMAP)) of 22,199 single nuclei transcriptomic profiles labeled by cell type. **B.** Representative markers for each major neuronal cell type in the hippocampus. **C.** Expression of cell type-specific marker genes for major neuronal populations. **D.** Nuclei displayed by group: vehicle (grey), corticosterone (orange), corticosterone + fluoxetine (purple), and corticosterone + ECS groups (green). **E.** Comparison of biological replicates following unsupervised clustering of hippocampal nuclei, including number of recovered nuclei per animal. **F.** Expression of granule cell specific markers for the total neuronal population and specifically for granule cells. **G.** Comparison of biological replicates following unsupervised clustering of granule cell nuclei, including number of recovered nuclei per animal.

**S4. Supplemental Figure 4**.


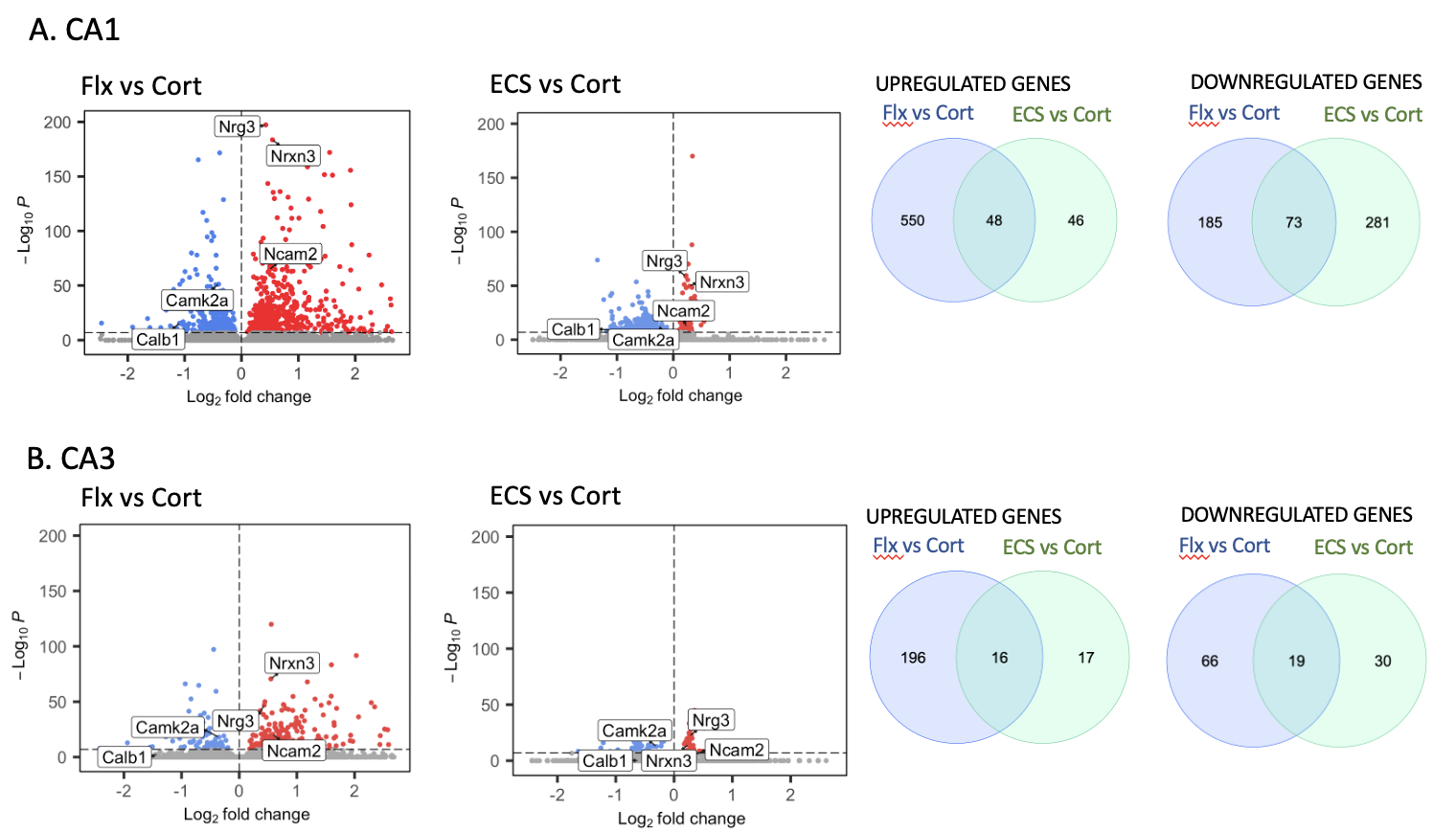


**S4. Supplemental Figure 4**. **Across all principal cells of the trisynaptic circuit, treatment with fluoxetine induces greater transcriptome upregulation while ECS induces a downward transcriptomic shift.** Volcano plots depicting expression level (-Log of the p value by Log2 fold change) of DEGs for Flx and ECS groups, vs Cort in CA1 **(A)** and CA3 **(B).** Venn diagrams depicting overlap of upregulated vs downregulated DEGs in Flx and ECS groups, vs Cort.
